# Do Specific Pedagogies and Problem-Based Teaching Improve Student Employability? A Cross-Sectional Survey of College Students

**DOI:** 10.3389/fpsyg.2020.01099

**Published:** 2020-06-30

**Authors:** Kerang Li, Michael Yao-Ping Peng, Zongmin Du, Jing Li, Ke-Tien Yen, Tsao Yu

**Affiliations:** ^1^School of History and Culture, Hebei Normal University, Shijiazhuang, China; ^2^School of Economics and Managemant, Foshan University, Foshan, China; ^3^School of Digital Economics, Guilin University of Electronic Technology, Guilin, China; ^4^Business School, University of National and World Economy, Sofia, Bulgaria; ^5^Dong Fureng Economic and Social Development School, Wuhan University, Wuhan, China; ^6^Department of Leisure and Sports Management, Cheng Shiu University, Kaohsiung, Taiwan

**Keywords:** pedagogy for employability, problem-based teaching mode, absorptive capacity, student employability, higher education

## Abstract

Higher education policy and manpower training have failed to meet the requirement of rapidly changing society and employers’ expectation in Taiwan, resulting in a significant gap between university education and employment. Student employability should also be a focus of all higher education institutions, although whether a high degree of student learning outcomes can represent a high degree of student employability is still unclear. This study explores the relationships among pedagogy for employability, the problem-based teaching mode, absorptive capacity, and student employability in higher education institutions (HEIs). Based on analysis of a total sample of 553 undergraduates from 16 Taiwanese HEIs using structural equation modeling, the results show that the influences of pedagogy for employability and the problem-based teaching mode on absorptive capacity and student employability are positively statistically significant. Based on the findings, specific suggestions and managerial implications for HEIs, curriculum and instruction planning, and future research are provided.

## Introduction

### Background

Higher education and its function in increasing human resources play a decisive role in the foundation of national economic development (Choi and Rhee, 2014). According to the statistics of each country, the number of higher education institutions (HEIs) has approached saturation and the education pattern has gradually transferred from elite education to mass education (Taylor et al., 2013; Ahmed et al., 2015). Although higher education reform policy has provided more educational opportunities for students, it has given rise to many other problems such as low teaching quality and competitiveness, which have become the latent problems of higher education development on a global perspective (Shin and Harman, 2009; Marginson, 2011). Recent studies on HEIs have pointed out that student learning outcomes can be significantly improved through teaching quality improvement, course setting reform, and resource and equipment optimization (Pike et al., 2011, 2012; Maringe and Sing, 2014). However, there are few studies investigating the link between student learning and student employment (Baek and Cho, 2018) from the perspective of the job market and the flow of talent. Students have developed many skills and abilities in tertiary institutions, showing a high degree of learning (Blázquez et al., 2018). This points back to a problem: a high degree of employability may have a high degree of learning outcomes, but a high degree of learning effectiveness cannot derive a high degree of employability. If students will eventually return to the job market, colleges should pay attention to student employment based on their training, rather than the improvement of student learning effectiveness. Therefore, this study aims to clarify the important role of student employability (SE).

### Research Purposes

De Vos et al. (2011) indicate that employability refers to an individual’s acquisition of knowledge, skills, and other characteristics in order to meet the needs of employers and reach professional potential (Vermeulen et al., 2018; Shahzad et al., 2020). As employability-related issues may differ under different research scenarios and designs, so do the research results and contributions (Bridgstock, 2009). Students should master knowledge and expertise at the university level and be able to apply their skills to a diverse workplace, to respond to employment requirements (Lurie and Garrett, 2017). Crossman and Clarke (2010) emphasize the link between the effectiveness of higher education and SE, which will affect the satisfaction of employers and graduating alumni (Bridgstock, 2009). After all, the characteristics and the visibility of HEIs are able to be improved through the performance of their students, which is able to improve or clarify employability (Franz, 2008; Hennemann and Liefner, 2010; Avramenko, 2012; Blázquez et al., 2018); that is, students may facilitate workplace trends, career exploration, career planning, career development, ethical values and work attitude, and even the development of employability, through the provision of relevant courses and the effort instructors put into reducing the gap between education and employment (Ahmed et al., 2015; Baek and Cho, 2018; Blázquez et al., 2018; Likisa, 2018). Instructors’ inspiration and guidance can improve students’ employability and increase their motivation for learning, input, and effectiveness (Robinson et al., 2008). By integrating well-defined employability activities in the classroom, instructors are able to promote a positive attitude toward learning and cultivate future employability. Based on these arguments, this study aims to explore how SE differs under various teaching modes.

In addition to employment-oriented teaching activities, there are many important learning modes conducive to the improvement of SE (Dunlap, 2005; Franz, 2008; Bridgstock, 2009). Through the application of improved learning modes, students can gain a better understanding of the circumstances in the real world (Hennemann and Liefner, 2010), and this enables instructors to analyze students’ problem-solving ability and achieve higher student learning outcomes (Pike et al., 2011, 2012; Choi and Rhee, 2014). Based on several scholars’ arguments, instructors also play an important role in designing the learning context (situations and outcomes) to enable students to achieve superior employability (Lent and Brown, 2006), the core of which is solving workplace-based problems. Therefore, instructor-driven problem-based learning (PBL) situations help students to invest in learning situations, acquire relevant resources/information search capabilities from the process of solving problems, acquire new knowledge to plan strategies, and enhance their learning effectiveness (Yorke and Knight, 2004; Spronken-Smith, 2005; Hmelo-Silver et al., 2007). Different from traditional teaching, PBL is student-centered self-orientation learning (Dunlap, 2005; Hmelo-Silver et al., 2007). It emphasizes the creative use of knowledge and the integration of theories and skills learned, and aims to cultivate logical reasoning and problem-solving ability (Hmelo-Silver and Barrows, 2006). The concept coincides with the development of SE. From research in recent years, it has been found that there are few studies of the effect of PBL on the learning tendency and problem-solving ability of college students. Therefore, the research on the role of PBL in the development of college students’ employability is of great value (Savery, 2006; Koh et al., 2008; Schmidt et al., 2011). Therefore, this study aims to, theoretically and practically, investigate the application of the problem-based teaching mode (PBTM) and its contribution to SE.

In students’ learning process, scholars have emphasized that individual behavior arises from the internal thought process of specific physiological or psychological goals (Jang et al., 2016; Shogren et al., 2018; Al-Jubari et al., 2019) in the form of “stimulus–response–enhancement” (Trenshaw et al., 2016), in which “enhancement,” mentioned in previous paragraphs, concerns SE. Zimmerman (2002) suggested that self-regulated learning in the literature showed that contemporary learning theory enables learners to explain the individual differences, from an emphasis on the ability of learners to prepare for these differences, toward a dynamic understanding of learning, and increasingly an emphasis on the establishment of learners’ self-adjustment ability. Similar to student absorptive capacity (AC) as emphasized by Fabrizio (2009), students should have a method of knowledge transformation to facilitate knowledge learning, digestion, transfer, and application to demonstrate knowledge efficiency (Shahzad et al., 2020). However, few studies have explored students’ absorption, digestion, and transfer of knowledge, especially the role of AC in the student learning process. Therefore, this study aims to verify the mediating role of AC between teaching modes and SE.

### Research Contributions

According to the above explanations, this study intends to propose relevant research contributions. Most related studies on SE have been database or qualitative research. This study adopts a questionnaire survey to investigate the formation of SE and training, collect relevant information, and understand students’ relation to the teaching model and internal AC in the change process. This study explores SE in the learning process in the form of “stimulus–response–enhancement” (Trenshaw et al., 2016), including pedagogy for employability (POE), PBL, AC, and SE. The results of the study can provide important results regarding the new directions and meanings of HEIs while rethinking the nature of education.

## Literature Review and Hypothesis Development

### Student Employability

In recent years, scholars have put more effort into employability-related research. The substantial technological, social, and economic changes that have occurred in recent decades (Abbas et al., 2015) have modified the concepts and operations of industrial organizations (Abbas and Sağsan, 2019) and HEIs across the world (Vermeulen et al., 2018). Hence, dynamic HEIs ensure the highest standards of human capital development, so that they can contribute to economic growth (Ahmed et al., 2015; Baek and Cho, 2018). Through research situations and design of methods, and the integration of theoretical and practical analysis, scholars have studied the meaning of employability and the causality between employability and other factors (Hennemann and Liefner, 2010; Avramenko, 2012; Baek and Cho, 2018). Van der Heijde and Van der Heijden (2006) have argued that employability is the individual’s appropriate application of competence (Blázquez et al., 2018), continuous acquisition and creation of essential work skills in order to accomplish all the tasks, and adaptation to internal and external labor market changes (Fugate et al., 2004; De Cuyper et al., 2008; Vermeulen et al., 2018; Shahzad et al., 2020). Hence, the need for critical and reflective thinking, problem-solving abilities, self-management, learning, and related competencies is continually increasing across all disciplines (Makkonen, 2017). Several prior studies have indicated that in addition to the influence of basic education on employability, factors like personal conditions, interpersonal relations, and external factors that cannot be acquired in higher education should also be considered (Ahmed et al., 2015; Cacciolatti et al., 2017; Blázquez et al., 2018).

Hennemann and Liefner (2010), who developed a graduate employability training process, summed up a comprehensive structure of impact factors to explain the capacity, capability, and competence (Blázquez et al., 2018) that are important elements in the process of developing employability (Lurie and Garrett, 2017; Likisa, 2018). Hennemann and Liefner (2010) distinguish among capacity, capability, and competence, and define “capacity” as the demonstration of one’s confidence in an uncertain environment, which could be built from experience; “capability” as the essence of future orientation, which can be regarded as the basic effect of an interactive learning process; and “competence” as learners with full confidence and the ability to show their consistent confidence in a wide range of situations (Hennemann and Liefner, 2010; Modestino et al., 2016; Makkonen, 2017; Blázquez et al., 2018). Nygaard et al. (2008) argue that knowledge, skills, and abilities should be included in university education and equip students with the ability to cover personal and social backgrounds (Kellermann, 2007; Blázquez et al., 2018), and make use of knowledge and skills through reflection.

On the other hand, the constituent elements of employability, such as national cultural conditions, industrial development, and population structure, should be taken into account (Ahmed et al., 2015; Cacciolatti et al., 2017; Blázquez et al., 2018; Vermeulen et al., 2018; Shahzad et al., 2020). The Department of Education (The Pedagogy for Employability Group, 2006) established an “employability skills framework” with eight categories: communication skills, teamwork ability, problem-solving ability, original and entrepreneurial ability, planning and organizational ability, self-management ability, autonomous learning, and scientific and technological ability. Pan and Lee (2011) surveyed the flow of HEI graduates in Taiwan adopting the employability scale developed by Andrews and Higson (2008). They suggested that employability should cover the general and professional ability required at work, work attitude, career planning ability, and confidence. This study adopts the employability classification of Pan and Lee (2011) as the measure of SE.

### Pedagogy for Employability

In the studies regarding student learning, scholars continually explore learning outcomes. They are based on a large degree of acquired knowledge, construction updates, deeper understanding, and discussion, and learners try to complete a specific task requiring basic foundational knowledge, especially by observing other people’s behavior (Oleson and Hora, 2014; Ahmed et al., 2015; Al-Jubari et al., 2019). Therefore, in the process of inspiring students’ learning orientation and engagement, teachers should abandon passive, teacher-oriented teaching methods, and adopt active, learner-centered activity design, and commit to encouraging students engaged in deeper understanding. Students can then apply real-life examples to different situations (Tagg, 2003; Al-Jubari et al., 2019). In addition, with the better network and connection with industry provided by the university, students should learn to acquire knowledge and skills for employment before entering the workplace (Corbett, 2005; Ahmed et al., 2015; Blázquez et al., 2018). Therefore, teachers should inspire students to learn the orientation and input process by using active, learner-centered activity design, and engage students in the deeper understanding and meaning of the subjects, so that students can apply the real-life paradigm of learning in different contexts (Tagg, 2003; Baek and Cho, 2018).

Yorke (2006) mentions in “Pedagogy for Employability” that the close correlation between higher education and the national economy has been accepted by governments around the world and reflects the importance of human capital perspectives (Becker, 1975; Baek and Cho, 2018; Blázquez et al., 2018). Reich (2002) and Reed (2002) also emphasize the economic benefits of creativity, entrepreneurship, and entrepreneurship in the workplace (Al-Jubari et al., 2019), showing that economic development not only reflects the quality of the factors, but also presents a series of skills development contexts. Therefore, the concept of an entrepreneurial spirit should be included in higher education to improve the discipline and students’ employment skills, understanding, quality, and positioning expectations.

Therefore, employment-oriented curriculum design and learning activities will meet the current concerns about student employment issues, and show that design is not a substitute, by demonstrating the relationship between employment-oriented teaching activities and professional disciplines (Ahmed et al., 2015; Baek and Cho, 2018). Yorke and Knight (2004) focus on teaching activities and employability embedded in curriculum design, providing teachers with the ability to effectively adjust the curriculum structure and improve it to provide better practice for POE (Baek and Cho, 2018). However, there are few studies that provide a clear measurement of employment-oriented teaching activities. Yorke (2006) proposes several designs for employment-oriented teaching activities, and attention should be paid to the principles of this study in order to clearly explore employment-oriented teaching activities as a measure for the current study, including analyzing case-study material; annotating a bibliography rather than writing “yet another essay”; writing critical commentaries, or reviews, perhaps in the style of a particular kind of publication; summarizing complex material in a short briefing paper or executive summary; constructing criteria against which performance might be judged; in-tray exercises, perhaps under time constraints; presenting a case, and being prepared to justify it; role-playing; group problem solving, including attention to the group dynamics of teamwork; and surveying the public’s perceptions, such as in collecting oral history data or consumer preferences.

In addition to social soft skills and hard skills, SE includes the psychological cognition and attitude of job seekers. Therefore, teachers should use internal and external inducements to guide students to improve their own employment conditions (Bogler et al., 2013). As general and professional ability represents students’ external learning output and academic performance, students need to reach a high degree of learning satisfaction to meet these conditions (Likisa, 2018). When students are satisfied with their learning situation, they are able to achieve better academic performance or learning outcomes (Bogler et al., 2013; Baek and Cho, 2018; Al-Jubari et al., 2019), which serve as a foundation for the development of employability. In class, teachers can design specific courses and implement their teaching content, methods, attitudes, and teacher–student interaction in the program (Corbett, 2005; Hmelo-Silver et al., 2007). Therefore, the study of employment-oriented teaching activities and the establishment of practical experience based on the application of the educational context help to explore the relationship between teaching activities and employability. The results will allow schools and teachers to understand the most appropriate curriculum planning and activities. For reasons outlined above, this study proposes the following hypotheses:

H1: POE will positively correlate to students’ AC.

H2: POE will positively correlate to students’ employability.

### Problem-Based Teaching Mode

The PBTM is a kind of learning model to which close attention has been paid in recent years (Savery, 2006; Koh et al., 2008; Schmidt et al., 2011). The model emphasizes students as the main focus of teaching, and divides the learning process into five stages: “asking questions–establishing hypotheses–collecting information–arguing hypotheses–summary.” It is also a learning environment that places students in a complex but meaningful problem situation that they then learn to solve through teamwork, acquiring knowledge through this process. Therefore they further develop problem-solving and self-learning ability (McGrath et al., 2006).

In the past, teachers had also used the PBTM to improve students’ exploration of knowledge (Chang et al., 2012). The study suggests that the characteristics of the PBTM are (1) student-centered learning patterns; (2) easier implementation with teacher-led and small-scale student groups; (3) teachers in the role of assistants or guides; (4) the discovery of the problem as an important key to gaining knowledge and solving the problem; and (5) self-guided learning as a better way to obtain more than necessary information and ability to solve problems when they occur (Hmelo-Silver et al., 2007; Chang et al., 2012). In other words, the PBTM is derived from students’ learning needs. Through the strategies of inquiry, collaboration, and reflection, teachers can improve students’ active participation, knowledge, and practical skills.

Previous studies show that the learning opportunities available to individuals have a positive effect on producing better performance and enhancing their self-efficacy (Zhao et al., 2005; Al-Jubari et al., 2019). Moreover, in order to reflect the impact of self-efficacy, students develop long-term appropriate learning experience, which would affect the environmental opportunities under the action (challenge) and personal ability (skills) subjective assessment (Likisa, 2018). When students encounter activities that require more knowledge and challenges, they will increase their individual input to overcome the challenges and receive an appropriate learning experience. Therefore, in addition to the internal incentives, the design of all kinds of learning activities should encourage students to explore the meaning of learning through the process of knowledge, shape their long-term learning goals (Delle Fave and Massimini, 2005), and provide a forecast for their future career (Bassi et al., 2007).

In the context of study, how students perceive self-efficacy could influence their academic interests, learning motivation, emotional management, cognitive ability, and achievement growth (Bandura et al., 1996; Bandura, 1997). Moreover, there is a strong intermediary effect on individual performance and self-practice in follow-up (Lent and Brown, 2006). Dunlap (2005) found that the PBTM helps students acquire the expertise and skills needed in the workplace. However, it is difficult to improve learning outcomes without self-efficacy as a prerequisite. Therefore, the teaching strategy of the PBTM should emphasize both short-term and long-term goal setting, and provide feedback regarding students’ learning performance as the source of learning and improving the setting, thus enhancing their self-efficacy. This study proposes hypothesis 3:

H3: PBTM will positively correlate to students’ AC.

Finally, the PBTM, with its relationship with SE, is helpful in improving students’ interest in learning and the application of their professional skills (Al-Jubari et al., 2019), and in further enhancing students’ capability (Spronken-Smith, 2005; Martin et al., 2008; Baek and Cho, 2018). When facing practical problems, such as critical analysis, problem solving, and reflection, students can demonstrate better learning attitudes and critical thinking ability. Duncan and Al-Nakeeb (2006) have confirmed that students who have accepted the problem-based teaching pattern will change their learning motives, attitudes, and behaviors so as to enhance their critical thinking, learning autonomy, and employment-related competencies (Baek and Cho, 2018). Therefore, this study proposes hypothesis 4:

H4: PBTM will positively correlate to students’ employability.

### Absorptive Capacity

Cohen and Levinthal (1990) proposed the concept of AC in 1990, which defined AC as “enterprises with the ability to identify new values, acquire external knowledge, and apply this knowledge for business purposes.” In other words, the organization’s AC is composed of three overlapping aspects: communication with the external environment, organizational knowledge level and experience, and the diversity of the knowledge structure (Likisa, 2018; Shahzad et al., 2020).

In the study by Nieto and Quevedo (2005), the definition of AC, combined with those of Cohen and Levinthal (1990) and Fiol and Lyles (1985), developed four aspects as the measurement of AC: communication with the external environment, organizational knowledge level and experience, the diversity of knowledge structure, and overlapping of strategic positions (Blázquez et al., 2018). Therefore, the organization has an excellent AC when it is able to absorb new knowledge and convert it into a product, process, or system. This kind of AC is based on both knowledge and the development of new knowledge and promotes the transfer of new knowledge (Abbas and Sağsan, 2019; Shahzad et al., 2020). Specifically, the savings of the organization’s own capability will determine how the organization applies, integrates, and even develops its core competencies (Blois, 1999; Likisa, 2018). Because a strong AC will allow the organization to acquire the idea of learning, even in the process of alliance or enterprise resource planning, AC is the implementation of knowledge transfer (Wang et al., 2007). If the organization has a higher degree of AC, the transfer of organizational knowledge will be rather effective (Boynton et al., 1994; Shahzad et al., 2020).

In contrast to the measurement of AC, Zahra and George (2002) have shown that AC represents the organization’s rules and procedures, which are achieved by acquisition, assimilation, transformation, and exploitation. This process will produce a dynamic organizational potential. The Tripsas (1997) study clearly states that AC is mainly for the identification and acquisition of external knowledge (Blázquez et al., 2018), and Zahra and George (2002) propose similar concepts.

Personal knowledge assimilation refers to individuals’ ability to analyze their external knowledge, interpretation, and understanding in their daily work (Szulanski, 1996). Zahra and George (2002) argue that knowledge assimilation includes interpretation, comprehension, and learning. From the point of view of external knowledge, Szulanski (1996) argues that individuals’ external knowledge is affected by the environment in which they live (Shahzad et al., 2020). In other words, external knowledge will differ in its meaning and value when used in different environments and thus enhance one’s understanding, digestion, and replication of the knowledge (Blázquez et al., 2018; Abbas and Sağsan, 2019). If the value of knowledge is dependent on existing complementary assets, it will make the knowledge more difficult to understand and apply.

Personal knowledge transformation ability refers to individuals’ ability to combine the newly absorbed knowledge with present knowledge. Individuals are able to interpret present knowledge by deleting the unnecessary parts and explaining it with a new perspective, according to their understanding of the new knowledge (Abbas and Sağsan, 2019). The transformation of knowledge comes from identifying two incompatible aspects of knowledge, combining it, and presenting it in new forms. Knowledge transfer capability can enhance the organization’s ability to gain insight into business opportunities (Smith and DeGregorio, 2002; Shahzad et al., 2020), help identify new opportunities, redefine the industry, and revise the organization’s competitive strategy (Christensen et al., 1998; Al-Jubari et al., 2019). For example, with business transactions and computer technology, two incompatible forms of knowledge, the network industry will combine these two and present it in a new form of e-commerce, known as the conversion of knowledge.

Personal knowledge exploitation means individuals’ ability to transform knowledge into the organization’s operation or innovation, and further revise, extend, and expand the existing competencies (Zahra and George, 2002). In the AC proposed by Cohen and Levinthal (1990), emphasis is placed on the application of organizational knowledge. Zahra and George (2002) argue that if the organization uses constructive, systematic, and sequential knowledge in operations, individuals in the organization will continue to produce new products, services, systems, processes, knowledge, and new organizational hierarchies (Shahzad et al., 2020).

Employability is composed of knowledge, technology, and diversity (Hennemann and Liefner, 2010; Baek and Cho, 2018). In the context of higher education, students may not have the knowledge and ability to absorb knowledge (Blázquez et al., 2018), even if the information provided by teachers is enriched. In other words, students with sufficient AC will be able to communicate and share knowledge about the connotation of knowledge through mutual interest and language, and then acquire valuable knowledge (Wenger, 1998; Cadiz et al., 2009; Abbas and Sağsan, 2019). Employability has a positive impact. Nor et al. (2012) also found that learning AC is good for students, meaning that they have prerequisite knowledge and academic performance. Therefore, they can effectively transfer knowledge and apply it to enhance their academic achievement and the development of their employability. According to the above description, this study deduces the following hypothesis:

H5: Students’ AC will positively correlate to employability.

## Methodology

### Research Framework

**FIGURE 1:**
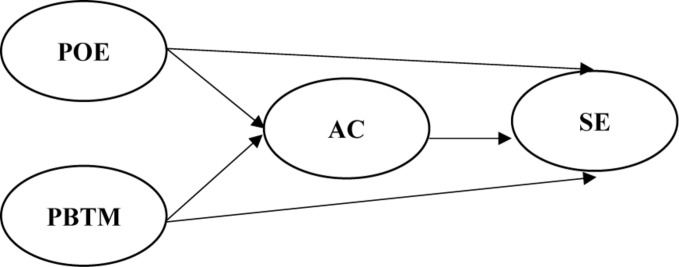
Research framework.

Based on the research motivations and the importance of the studies, this study summarizes SE-related studies conducted in the past. The research framework shown in Figure 1 displays the correlation between variables.

### Variable Measurement

Employability is a kind of social psychological construction, which includes subjective and objective aspects (De Vos et al., 2011). It can help employees respond to the demands of the employment environment and adjust their characteristics, behaviors, cognitions, and emotions, thus maintaining their adaptability and flexibility in the workplace. In order to learn about employees’ cognition of employability, this study includes “General Ability for Work” (GAW) (8 items), “Professional Ability for Work” (PAW) (4 items), “Attitude at Work” (AW) (3 items), and “Career Planning and Confidence” (CPC) (3 items), which were summarized from the research of the Australian Chamber of Commerce and Industry (2006) and the variable measurement of Pan and Lee (2011). The content covers 18 subjects: expression and communication, time management, leadership, innovation, team work, native language, foreign language, stability and pressure resistance, professional knowledge and skill, computer literacy, application of theory to work, problem finding and solving, learning desire, plasticity, understanding of professional ethics, understanding and planning of individual career development, understanding of environment and development of industries, and job search and self-promotion.

In the “Problem-Based Teaching Mode” section, the study is defined as a student-centered, guided teaching strategy that allow students to share knowledge and resolve problems through group learning processes and therefore enhance their problem-solving skills. In this study, the scale of the PBTM was developed using Chen et al. (2006) and Chang et al. (2012), including “Knowledge Sharing” (KS) (3 items) and “Problem Solving” (PS) (3 items) to measure students’ awareness of the PBTM.

In the “Pedagogy for Employability” (POE) section, this study is defined as teaching programs that are committed to fostering students’ ability to enter the workplace in the future. In this study, the teaching items proposed by Pegg et al. (2012) were converted into a measurable scale to measure the perceived level of POE.

In the “Absorptive Capacity” section, in order to understand the degree of awareness of students’ absorptive capacity (AC), this study defines the AC system as able to identify new knowledge by identifying (identifying and filtering valuable information), digesting (transforming new knowledge into applicable knowledge), and applying (using knowledge) the process into the knowledge available. The questionnaire was designed using Cadiz et al. (2009), including Assessment (3 items), Assimilation (3 items), and Application (3 items). Respondents had to indicate to what extent they agreed with statements on a seven-point Likert scale (1 = totally disagree; 7 = totally agree). All scales are shown in Appendix 1.

### Sampling

This study proposed a framework to explore the correlations and development model of PBTM, POE, AC, and SE. It sampled Taiwanese HEIs, including public, private, vocational, and general institutions. Of the sampled representative HEIs in Taiwan, there were a total of seven national HEIs and nine private HEIs. To confirm whether there were differences in the responses between national and private HEIs, this study conducted a non-response deviation test. The results of an independent sample *t*-test show that there are no significant differences in the basic data of the main aspects, which means no significant differences among the sample data of these HEIs.

This study selected 16 Taiwanese HEIs and sent 1000 questionnaires to them. After simple random sampling, a total of 563 questionnaires were returned, with an effective response rate of 56.3%. Since freshmen were not familiar with the learning environment, all participants in this study were sophomores, junior and senior students. This study obtained 553 valid questionnaires, excluding 10 invalid answers, and 56.6% were from females.

## Analysis and Results

### Reliability and Validity

This study adopted structural equation modeling (SEM) for the analysis. All scales were reliable, with composite reliabilities ranging from 0.82 to 0.95, all of which exceeded the benchmark of 0.70. Table 1 shows the reliability for each scale and factor loadings for each item. Confirmatory factor analysis was examined with LISREL 8.54 to verify the convergent and discriminant construct validities of the scales (Anderson and Gerbing, 1988). Hair et al. (2006) recommend that convergent validity should be assessed using three indicators: (1) standardized factor loading higher than 0.70; (2) average variance extracted (AVE) above 0.50; and (3) composite reliability above 0.70. The evaluation standard for discriminant validity is the square root of AVE for one dimension and should be greater than the correlation coefficient with any other dimension(s). Table 1 shows that all three criteria were met, except for the slightly lower AVEs of role identity, integrative learning, and cognitive gains. The correlation coefficients of the dimensions were all less than the square root of the AVEs, suggesting that each had good discriminant validity.

**TABLE 1:** Measurement.

	1	2	3	4	5	6	7	8	9	10
(1) GAW	***0.69***									
(2) PAW	0.840**	***0.80***								
(3) AW	0.789**	0.762**	***0.79***							
(4) CPC	0.786**	0.762**	0.757**	***0.83***						
(5) POE	0.687**	0.650**	0.678**	0.700**	***0.81***					
(6) Assessment	0.584**	0.563**	0.468**	0.473**	0.557**	***0.94***				
(7) Assimilation	0.500**	0.525**	0.425**	0.428**	0.543**	0.854**	***0.88***			
(8) Application	0.564**	0.523**	0.459**	0.456**	0.627**	0.868**	0.869**	***0.89***		
(9) KS	0.533**	0.522**	0.489**	0.485**	0.553**	0.742**	0.717**	0.737**	***0.91***	
(10) PS	0.479**	0.482**	0.414**	0.395**	0.559**	0.775**	0.818**	0.819**	0.819**	***0.88***
Means	5.67	5.58	5.65	5.46	5.51	5.61	5.86	5.62	5.32	5.85
SD	1.22	1.34	1.33	1.54	1.35	1.68	1.80	1.73	1.85	1.76
Cronbach’s α	0.87	0.88	0.82	0.86	0.95	0.93	0.91	0.92	0.93	0.91
AVE	0.47	0.64	0.63	0.68	0.65	0.88	0.78	0.80	0.82	0.77
CR	0.88	0.88	0.83	0.87	0.95	0.93	0.92	0.92	0.93	0.91

**p < 0.01.

### Examining Fit Indexes of Structural Model

In this study, the modality of the model was verified by SEM, and the model was used to evaluate the model with the seven indicators (Jöreskog and Sörbom, 1993). Results are shown in Table 2 the modality of this model should be acceptable.

**TABLE 2:** Fit indexes result of structural model.

Index	Standard	Results
χ^2^/degree	<3.00	2.259
Goodness of fit index, GFI	>0.8	0.892
Adjusted goodness of fit index, AGFI	>0.8	0.838
Root mean square error of approximation, RMSEA	<0.08	0.084
Normed fit index, NFI	>0.9	0.945
Comparative fit index, CFI	>0.9	0.968
Incremental fit index, IFI	>0.9	0.968

### Hypothesis Testing

**FIGURE 2:**
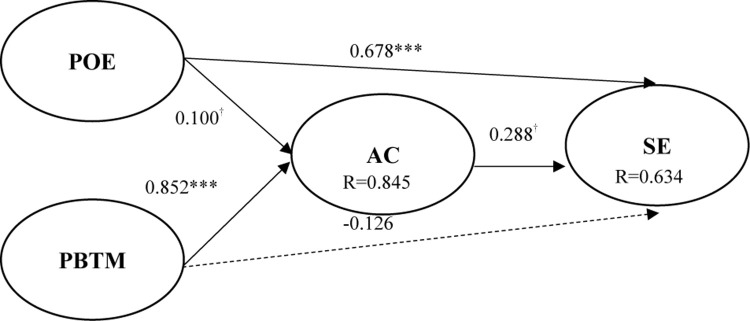
Path analysis. ^†^*p* < 0.1; **p < 0.01.

The study verified relationships among constructs via SEM. For constructs with a higher-order factor structure (PBTM, AC, and SE) that are formative in nature, Lee and Cadogan (2013) suggest that researchers should avoid developing and assessing a model containing a direct link from the antecedent variable to the aggregate endogenous variable. Therefore, the author reduced the number of parameters to be estimated following the partial aggregation method (Little et al., 2002). The structural model is shown in Figure 2.

H1 and H2 state that POE positively affects students’ AC and employability. Figure 2 shows that POE (*t* = 1.794, *p* < 0.1; *t* = 8.824, *p* < 0.001) has significantly positive influences on students’ AC and SE, which supports H1 and H2. It shows that the higher the degree of instructional activity of the teacher, the better the AC and SE of the student. Moreover, this study further deduces that PBTM has a positive effect on AC and SE and the results show that PBTM (*t* = 13.213, *p* < 0.001; *t* = −0.732, *p* > 0.1) is helpful by providing problem-oriented teaching activities and by assisting in improving students’ AC (*t* = 1.690, *p* < 0.1) has significantly positive effects on SE, which supports H5, which means a higher degree of student AC. Its employment capacity has a positive correlation with the upgrade of the teaching model.

## Discussion and Implications

### Conclusion

The results show that POE has positive impacts on students’ AC and employability. At present, the proportion of Taiwanese HEIs with a focus on employment remains low. Many teachers still adopt the traditional pattern and style of curriculum design and teaching activities. They are still quite familiar with the connections among industries. Most universities are beginning to transform and strengthen, however. Although there is a comprehensive understanding of the links between industries, there is no clear understanding of how to carry out practical teaching/case-study learning and conditions for the promotion of teachers’ employment-oriented teaching activities. This study suggests that teachers should understand conditions and standards when designing curricula and teaching activities to improve SE and achieve practical teaching/case-study learning.

Furthermore, our research findings show no significant correlation between PBTM and SE; instead, there may even be a negative effect. However, there is a strong positive effect on students’ AC and plays an important intermediary role in PBTM and SE. PBTM focuses on students’ ability to effectively use their own knowledge to solve problems and obtain new knowledge through this process. If PBTM fails to allow students to absorb knowledge, students may have difficulty engaging in the teaching situation or even applying the knowledge and practice obtained in their future workplace. Therefore, this study suggests that teachers should reorganize the space and time for learning when designing a curriculum and teaching activities, so that students can rethink the elements during the process of problem solving, and further help them assimilate, convert, absorb, and understand their application.

In addition, the results pertaining to the path relationship of the model in this study indicate that AC has a positive and significant influence on SE. This finding shows that students’ engagement in various knowledge learning activities increases to improve their AC when they are fully aware of changing global competition and employment markets from PBTM and POE. Accordingly, our results support the notion that AC plays an important transformational role among motivation, attitudes, and SE in a complete learning process.

### Discussion

This study aims to explore the curriculum- and teaching-related literature and better understand the concept of SE, its nature and meaning. The focus of previous studies on employability was on the level of the employment ability of enterprises (Modestino, 2016; Baek and Cho, 2018; Abbas and Sağsan, 2019). However, few studies focused on the employment ability of HEIs through teachers’ unique employment-oriented teaching curriculum (Cacciolatti et al., 2017; Likisa, 2018). Based on the curriculum and teaching view, the source of formation of SE is discussed combined with different theoretical aspects (Makkonen, 2017) to make the process of formation more complete.

Therefore, this study investigated this area, combined with the curriculum, teaching, and “external stimulus–internal attribution–behavior” model, and hoped to gain more theoretical knowledge regarding the academic performance of students via academic research. This study found that POE has a positive impact on students’ AC and employability. This result was similar to those of Corbett (2005); Hmelo-Silver et al. (2007), and Lent and Brown (2006). Therefore, teachers should incorporate the competency needed to develop students’ future employment when they are designing courses and teaching models.

Furthermore, another interesting finding is that PBTM does not have a significant impact on SE. The results echo Zimmerman’s (2002) self-regulated learning theory and Hennemann and Liefner’s (2010) employability building model, which both indicate that SE should follow a development path based on simulation of the teaching and learning context to improve students’ skills and capabilities, and further formulate their employability.

Although this study has not discussed the mediating mechanism of AC on the relationships among PBTM, POE, and SE, the results show that students’ higher awareness of PBTM and POE make it easier to lead to a knowledge learning process of AC with high intensity, and this intensified AC will contribute to SE. This conclusion is consistent with the findings of Wang et al. (2007); Cadiz et al. (2009), and Nor et al. (2012) that AC is established over time, and the stronger AC students have, the more effectively they can exploit the knowledge obtained (Shahzad et al., 2020), which indicates that the influence of AC on the development of SE cannot be ignored.

### Teaching Implications

This study found that POE has a positive impact on students’ AC and employability. This result suggests that teachers should integrate students’ ability into teaching materials to develop the elements critical in future employment when designing course content and teaching models, instead of overemphasizing the fixed recitation of the course and teaching. At present, while most universities are taking part in transforming and strengthening the link between industry and the classroom, how to increase teachers’ capacity to design pedagogy by adding employability-related materials into the teaching system remains unclear. This study suggests that teachers should understand the conditions and standards of the curriculum and teaching activities to equip students with important employability skills and therefore successfully create effective learning. Problem-oriented teaching activities focus on students who can effectively apply their own knowledge to solve problems and further learn new knowledge from the process. If problem-solving teaching activities fail to allow students to absorb knowledge, students may have difficulty engaging in the teaching situation or even applying the knowledge and practice obtained in the future workplace. Therefore, this study suggests that teachers should reorganize the space and time for learning when designing the curriculum and teaching activities so that students can rethink the elements during the process of problem solving, and further help them assimilate, convert, absorb, and understand their application.

This study suggests that teachers’ design of curriculum content and the teaching model should be combined with practice, diverse activities, and case studies to promote students in a bid to develop their positive attitude toward academic learning, which will increase students’ involvement and thus improve their knowledge. Teachers should pay extra attention to motivating students to learn. In this study, the results show that students who participate in case discussion and results sharing are more willing to challenge themselves, invest actively, and build stronger self-confidence. Moreover, the combination of teaching and student inquiry activities will motivate students’ active learning and develop their employability.

### Research Limitations and Suggestions for Further Studies

Scholars believe that students will be able to analyze the differences between their learning outcomes and learning inputs, such as gender, economic income, weekly study time, and so on. Pike et al. (2006) mention that previous studies have examined student performance at different points in time, such as freshmen, sophomore, junior, and senior years. However, putting participants from different academic years into a single analysis may result in errors. That is, the relationship between the variables, the characteristics of students with different learning styles and learning different problems, may not be able to yield correct results. It is suggested that future research should control the relevant important variables that may influence the results of the study, or analyze the differences between these variables to gain a better understanding of the effectiveness of the case teaching strategies with different traits.

Moreover, this study required students to self-report details on their learning awareness and knowledge learning as the employability indicator, mainly because actual SE data is confidential and not easily obtained. However, errors may exist in the students’ self-statement of their employability. The link between learning activities and SE may be better understood if students’ actual employability is assessed, with due consideration for research ethics. Furthermore, this study suggests that future researchers include interview content, students’ observations of learning status, and other qualitative data collection methods in their studies to support the research results and make a comprehensive judgment. Also, it is suggested that different independent variables or related situational variables could be added to enhance students’ perceived employability in future research.

Finally, while studies using learning theory have achieved remarkable results and made significant contributions in the field of higher education, few of them discuss the relationship between student learning outcomes and employability. This study has established an employability development framework with the adoption of a competence enhancement model, and the results have provided valuable insights into student learning theory. However, there are other motivational theories that may explain how to improve SE, such as attribution theory, self-efficacy theory, and need-hierarchy theory. Therefore, future studies could adopt these models to construct the dimensions that may influence employability.

## Author’s Note

Manuscript which first appeared as conference paper (Peng et al. (2017), “The Study on Application of Big Data Analysis to Improve Student Employability: Teaching Modes as Antecedent Variables,” in proceedings of 2017th Annual SEAAIR Conference in Singapore, 2017, SEAAIR, Singapore, pp. 405–426.) in 2017th Annual SEAAIR Conference can be considered as original work. This Manuscript has been revised and expanded.

## Data Availability Statement

The raw data supporting the conclusions of this article will be made available by the authors, without undue reservation, to any qualified researcher.

## Author Contributions

KL and MP contributed to the ideas of educational research, collection of data, and empirical analysis. MP, ZD, and K-TY contributed to the data analysis, design of research methods, and tables. TY and JL participated in developing a research design, writing, and interpreting the analysis. All the authors contributed to the literature review and conclusions.

## Conflict of Interest

The authors declare that the research was conducted in the absence of any commercial or financial relationships that could be construed as a potential conflict of interest.

## Appendix 1 Scales

**Table:**

Construct	Variables	Items
Student Employability	General ability for work	Expression and communication
		Time management
		Leadership
		Innovation
		Team work
		Native language
		Foreign language
		Stability and pressure resistance
	Professional ability for work	Professional knowledge and skill
		Computer literacy
		Application of theory to work
		Problem finding and solving
	Attitude at work	Learning desire
		Plasticity
		Understanding of professional ethics
	Career planning and confidence	Understanding and planning of individual career development
		Understanding of environment and development of industries
		Job search and self-promotion
Problem-based teaching model	Knowledge sharing	Organized and prepared for small group sessions.
		To share thoughts and opinions with peer actively.
		To share all sources for picture, text, and other information.
	Problem solving	Utilizes relevant resource materials effectively.
		Utilizes internet or evidence-based materials to get appropriate information.
		Applies knowledge to new situations to solve problems and to reach decisions.
Pedagogy for Employability	Pedagogy for Employability	Analyze a case data
		Annotate a bibliography
		Write critical reviews or tips
		Simplify complex data into summary reports
		Construct the evaluation criteria for learning outcome
		Do exercise within limited time
		Report a case and prepare a defense
		Proper role play
		Solve problems through teamwork
		Investigate public perceptions and trends (such as collecting data or public preferences)
Absorptive capacity	Assessment	I am able to decipher the knowledge that will be most valuable to me.
		It is easy to decide what information will be most useful in meeting my course.
		I know enough about the knowledge I use to determine what new information is credible and trustworthy
	Assimilation	The shared knowledge within my class makes it easy to understand new material presented within our technical areas.
		It is easy to see the connections among the pieces of knowledge held jointly within my class.
		Many of the new knowledge developments coming to the class fit well into the current knowledge.
	Application	It is easy to adapt my work to make use of the new major knowledge made available to me.
		New major knowledge can be quickly applied to my work.
		My peers can immediately benefit from new major knowledge learned in the class.
