# Corrigendum: Do Specific Pedagogies and Problem-Based Teaching Improve Student Employability? A Cross-Sectional Survey of College Students

**DOI:** 10.3389/fpsyg.2020.01872

**Published:** 2020-08-12

**Authors:** Kerang Li, Michael Yao-Ping Peng, Zongmin Du, Jing Li, Ke-Tien Yen, Tsao Yu

**Affiliations:** ^1^School of History and Culture, Hebei Normal University, Shijiazhuang, China; ^2^School of Economics and Management, Foshan University, Foshan, China; ^3^School of Digital Economics, Guilin University of Electronic Technology, Guilin, China; ^4^Business School, University of National and World Economy, Sofia, Bulgaria; ^5^Dong Fureng Economic and Social Development School, Wuhan University, Wuhan, China; ^6^Department of Leisure and Sports Management, Cheng Shiu University, Kaohsiung, Taiwan

**Keywords:** pedagogy for employability, problem-based teaching mode, absorptive capacity, student employability, higher education

In the published article, there was an error in the affiliation of Jing Li. Instead of “Department of Leisure and Sports Management, Cheng Shiu University, Kaohsiung, Taiwan,” it should be “Dong Fureng Economic and Social Development School, Wuhan University, Wuhan, China.”

The authors apologize for this error and state that this does not change the scientific conclusions of the article in any way. The original article has been updated.

